# Identifying longitudinal patterns of CPAP treatment in OSA using growth mixture modeling: Disease characteristics and psychological determinants

**DOI:** 10.3389/fneur.2022.1063461

**Published:** 2022-11-17

**Authors:** Huijie Yi, Xiaosong Dong, Shaomei Shang, Chi Zhang, Liyue Xu, Fang Han

**Affiliations:** ^1^Department of Respiratory and Sleep Medicine, Peking University People's Hospital, Beijing, China; ^2^School of Nursing, Peking University, Beijing, China

**Keywords:** obstructive sleep apnea, continuous positive airway pressure, adherence patterns, growth mixture modeling, psychological characteristics, daytime sleepiness, daytime dysfunction

## Abstract

In this study, we aim to identify the distinct subtypes of continuous positive airway pressure (CPAP) user profiles based on the telemedicine management platform and to determine clinical and psychological predictors of various patterns of adherence. A total of 301 patients used auto-CPAP (Autoset 10, Resmed Inc.) during the treatment period. Four categories of potential predictors for CPAP adherence were examined: (1) demographic and clinical characteristics, (2) disease severity and comorbidities, (3) sleep-related health issues, and (4) psychological evaluation. Then, growth mixture modeling was conducted using Mplus 8.0 to identify the unique trajectories of adherence over time. Adherence data were collected from the telemedicine management platform (Airview, Resmed Inc.) during the treatment. Three novel subgroups were identified and labeled “adherers” (53.8% of samples, intercept = 385, slope = −51, high mean value, negative slope and moderate decline), “Improvers” (18.6%, intercept = 256, slope = 50, moderate mean value, positive slope and moderate growth) and “non-adherers” (27.6%, intercept = 176, slope = −31, low mean value, negative slope and slight decline). The comorbidities associated with OSA and the apnea–hypopnea index (AHI), which reflects the objective severity of the disease, did not differ significantly among the subgroups. However, “improvers” showed higher levels of daytime sleepiness (8.1 ± 6.0 vs. 12.1 ± 7.0 vs. 8.0 ± 6.1 in SWIFT, *p* = 0.01), reduced daytime function (4.6 ± 1.6 vs. 3.8 ± 1.6 vs. 4.2 ± 1.8 in QSQ daytime symptoms, *p* = 0.02), and characteristics of positive coping style (1.8 ± 0.5 vs. 1.9 ± 0.5 vs. 1.7 ± 0.5 in SCSQ positive coping index, *p* = 0.02). Negative emotion was more pronounced in patients with “non-adherers” (12.9 ± 3.8 vs. 13.7 ± 3.3 vs. 14.6 ± 3.5, *p* = 0.02 in the HADS depression dimension; 9.0 ± 6.1 vs. 9.8 ± 5.1 vs. 11.5 ± 6.3, *p* = 0.01 with Negative Affectivity in DS14, and 9.3 ± 6.1 vs. 10.3 ± 5.1 vs. 11.7 ± 6.5, *p* = 0.01 with Social Inhibition in DS14). Overall, our study demonstrated that CPAP therapy may present distinct trajectories of adherence over time in addition to the traditional binary classification. Self-reported sleep health issues (diurnal sleepiness and daytime dysfunction) as well as psychological characteristics (negative emotions and coping style) were predictors of different adherence subtypes in patients with OSA. Understanding CPAP use profiles and their predictors enable the identification of those who may require additional intervention to improve adherence and further enhance the therapeutic effect in OSA patients.

## Introduction

Obstructive sleep apnea (OSA) is a sleep-related breathing disorder characterized by repetitive collapse of the upper airway resulting in episodic oxygen desaturations, sleep arousal, and excessive daytime sleepiness (1–3). Patients with untreated OSA are at greatly increased risk for neurocognitive decline, hypertension, cardiovascular disease, and stroke (4, 5). Continuous positive airway pressure treatment (CPAP) of OSA has been shown to be effective in reducing these risks (6–8). However, adherence to CPAP therapy, which refers to the duration of mean nightly usage, is the greatest barrier to the effective treatment of OSA (9). The threshold of 4 h per night of CPAP administered during 70% of the days monitored has been widely predefined to meet minimum standards of good adherence (10). The first study described objective patterns in adherence to CPAP therapy in OSA patients by applying a CPAP machine containing a microprocessor and monitor, and only 46% of patients met this criterion for regular use (11). However, investigators have suggested that this methodological threshold of adherence fails to take into account individual differences in patterns of adherence, thus limiting the ability of researchers to understand the complex nature of adherence behavior. Weaver et al. explored the night-to-night variability and variation in the 1st week duration of CPAP treatment and defined two use patterns named consistent user and intermittent user (12). This was the first study to emphasize the trajectories of adherence over time. However, the description of adherence continues the traditional binary classifications of good or poor, and statistical techniques have never been employed to explore multiple characterizations of patterns with CPAP use.

Recently, several approaches have been used to evaluate profiles of CPAP use over time, which described a long-term time-series trend of adherence. Aloia et al. extracted the CPAP treatment data of 71 patients with OSA and identified seven different adherence trajectories using time series analysis (13). Another study involved a sample of 161 participants. Four longitudinal patterns (great users, good users, low users, and slow decliners) of treatment subgroups were identified by time series analysis combined with dynamic cluster analysis (14). However, this approach requires a sufficient amount of data, such as the daily night usage time of CPAP with OSA patients, and the sensitivity to mean changes with dynamic cluster analysis is weak, which may result in a lack of recognition among subtypes. In addition, the complicated procedure for identifying the user subtypes may reduce the practicality of this approach in clinical work. One study conducted by Sampaio et al. (15) optimized the traditional dichotomies of previous adherence patterns and created a usage gradient named “great adherent,” “moderately adherent,” and “poorly adherent.” Some studies have obtained three similar categories using an empirically driven analysis approach (16, 17). In Wohlgemuth's study, three CPAP user profiles labeled “non-adherers,” “attempters,” and “adherers” were identified based on the adherence data of the cross-section of the last follow-up node using latent profile analysis (LPA). Different from LPA, growth mixture modeling (GMM) is used to examine unique longitudinal trajectories with repeated follow-up measures. With the advantages of capturing inter-and-intra-individual differences over time, GMM has been extensively used to identify patterns of medication non-adherence. However, the longitudinal changing trajectories of adherence to CPAP treatment need to be further investigated.

Refined classifications of adherence changing patterns may contribute to deeply exploring the predictive factors affecting adherence and guide clinical intervention. To date, studies targeting predictors may still focus on binary classifications of adherence (18, 19). Factors that have been identified included the following overlapping domains: disease-related characteristics (20–22), psychological or behavioral factors (23), and technical/equipment-related factors. Severe daytime sleepiness has been shown to be an independent predictor of long-term good adherence in OSA patients (24). In addition, psychological traits such as depression and anxiety emotions in OSA patients have also been increasingly noticed by researchers to be a major obstacle to CPAP use (25–27). A better understanding of CPAP usage profiles makes it possible to distinguish the predictors of adherence patterns, particularly those amenable to interventions, so as to promote the healthy behavior of regular CPAP use.

In this study, our primary aim was to provide a description of the adherence profile of CPAP users with OSA using outcome variables from the CPAP treatment telemedicine management platform. We applied growth mixture modeling to evaluate the distinct subtypes of CPAP users. Second, we included covariates in our analysis to explore the relationship between baseline characteristics and adherence patterns of OSA patients. Thus, interventions targeting specific subtypes may be figured out to increase the possibility of transferring non-adherents to adherents and enhance the therapeutic effect of CPAP with OSA patients.

## Methods

### Participants

The sample consisted of 301 patients who were newly diagnosed patients with OSA and prescribed CPAP treatment aged 18 years or more at Peking University People's Hospital from March 2019 to May 2022. According to the diagnosis and treatment guidelines of OSA recommended by the American Academy of Sleep Medicine (AASM), patients with apnea hypopnea index (AHI) ≥5 times/h combined with severe symptoms (sleepiness, cognitive dysfunction), or AHI > 15 times/h were recruited and assigned to CPAP treatment. All patients underwent a standardized telemedicine diagnosis and treatment protocol that included general clinical evaluation, home sleep test, questionnaires evaluation of disease symptoms and psychological traits and CPAP treatment. During the period of CPAP treatment, sleep physicians will review the adherence data of CPAP treatment for OSA patients *via* a telemedicine management platform at 1 week, 1 month, and 3 months and follow up by telephone. Sleep specialists evaluated the response of participants to CPAP treatment, asked if there were obstacles restricting CPAP use, and provided suggestions on how to overcome these problems. In addition, participants were asked to complete questionnaires evaluating the improvements in symptoms after treatment at 1 month. This research was approved by the Medical Ethics Committee of Peking University People's Hospital (2015PHB187-01).

### Procedures

#### Sleep test

The type 3 portable monitoring device NOXT3 (Nox Medical, Reykjavik, Iceland) was used to diagnose OSA in our study, which records nasal airflow, thorax and abdominal respiratory efforts, pulse oximetry, air pressure, and body position (28). The scoring of sleep tests was completed by sleep technologists in accordance with the American Academy of Sleep Medicine (AASM). Apnea was defined as a decrease of ≥90% in the peak value of respiratory airflow signal compared with the baseline and a decrease duration of ≥10 s. Hypopnea was defined as a decrease of ≥30% in the peak value of respiratory airflow signal compared with the baseline and a decrease of ≥3% in oxygen saturation (29). The apnea hypopnea index (AHI) is the number of respiratory events per hour in the HSAT. According to the international classification of sleep disorders, patients with AHI ≥5 events/h and OSA-related symptoms such as sleepiness, hypertension or type 2 diabetes were diagnosed with OSA (30).

#### CPAP treatment initiation

The participants diagnosed with OSA received CPAP (Autoset 10™ Plus C, Resmed, Australia) and underwent a standardized, 30-min mask fitting and equipment educational session conducted by the CPAP technician. The sleep physician helped choose a mask (migrate FX, Resmed, Australia) and introduced the working principle of CPAP, the benefits of treatment, and how to avoid and deal with possible side effects. The CPAP pressure setting was 6–20 cm H_2_O. All Resmed S10 CPAP devices have a mobile communication chip that connects to the telemedicine monitoring cloud platform, which transmits CPAP treatment data in real time automatically. Patient adherence and therapy data are uploaded to the platform daily, typically within 1 h after each therapy session. It enables healthcare professionals to quickly access patient data, share clinical insights with other health professionals, and help patients solve problems during ventilator treatment in time. Sleep physicians will help patients complete their registration on the platform. The information provided includes usage data (hours of daily use and days used per month) and therapy issues (e.g., therapeutic modalities, mask leak, residual AHI, and type of apnea). Residual AHI is defined by an apnea-hypopnea index after CPAP therapy (the normal residual AHI ≤ 5 events/h).

### Outcome measures

Demographic characteristics, comorbidities, self-reported disease severity, and psychological assessment were recorded at baseline. Self-reported daytime sleepiness, fatigue, and insomnia were measured using the Epworth Sleepiness Scale (ESS), Sleepiness-Wakefulness Inability and Fatigue Test (SWIFT), and Insomnia Severity Index (ISI). The impact of daytime sleepiness on activities of daily living was measured by the Functional Outcomes of Sleep Questionnaire-10 (FOSQ-10), Pittsburgh Sleep Quality Index (PSQI), and Quebec Sleep Questionnaire (QSQ). Psychological assessment mainly included anxiety and depression symptoms, personality, and familiar coping characteristics in OSA patients, using the Hospital Anxiety and Depression Scale (HADS), Type D Personality Scale-14 (DS-14), and Simplified Coping Style Questionnaire (SCSQ). The use of CPAP was recorded from the telemedicine monitoring cloud platform.

#### Questionnaires

(1) ESS: ESS was developed to assess the degree of daytime sleepiness of patients. The instrument has eight items. The probability of dozing in each question is “never,” “mild,” “moderate,” and “severe.” The scores are 0, 1, 2, and 3. The maximum total score is 24. If the total score is more than 9, the patient is considered to have daytime sleepiness and risk of OSA. The split-half reliability coefficient and Cronbach's alpha coefficients of the Chinese version of the ESS were 0.81 and 0.80, respectively (31).
(2) SWIFT: the SWIFT is 12-item questionnaire with two subscales measuring sleepiness-wakefulness inability and fatigue. Subscale A involves six questions related to difficulty staying awake/wakefulness inability in different situations that might affect performance or cause adverse consequences; Subscale B has six questions related to fatigue, tiredness, or lack of energy in different situations. All scales require a 4-level (0–3) Likert response, and higher scores indicate lower wakefulness, higher fatigue, or lack of energy (32).
(3) ISI: The ISI assesses the subjective severity of insomnia in the past 2 weeks. It consists of seven items, each of which has a score of 0–4, with higher scores indicating more severe insomnia. The Chinese version of the ISI was verified by Chung et al. (33), and Cronbach's alpha coefficients and retest reliability were 0.83 and 0.79, respectively.
(4) FOSQ-10: FOSQ-10 was adapted by Weaver in 2009 (34). By selecting the items of the original FOSQ-30, 10 items and 5 dimensions were finally determined, including general condition (two items), activity level (three items), vigilance (three items), social outcome (one item), and intimacy and sexual relations (one item). Each item is set with four options of “very difficult, moderately difficult, somewhat difficult, and no difficult,” and the score is scored on a scale of 1–4. The total score is the average score of each dimension multiplied by 5, and the total score is 20. The higher the score, the better the daytime functional status of OSA patients. The Cronbach's alpha coefficient of the scale was 0.84, and the test-retest reliability was 0.73.
(5) QSQ: The QSQ is a specific quality of life scale for OSA that is designed to evaluate the effect of clinical treatment. The QSQ is a self-rating scale with 5 dimensions and 32 items, including daytime sleepiness, daytime symptoms, nighttime symptoms, mood changes, and social interaction. The item score is 1–7 using the Likert 7-point scoring method. The score of each dimension is equal to the sum of the scores of items in the dimension divided by the number of items in the dimension. The total score of QSQ is the sum of the mean scores of the five dimensions divided by 5. The higher the score, the better the quality of life (35).
(6) PSQI: The PSQI evaluates seven domains: (a) sleep quality; (b) latency; (c) duration; (d) habitual sleep efficiency; (e) use of medications; (f) disturbance; and (g) daytime dysfunction. The scores from these domains can be summed to produce a global score. The total score ranges from 0 to 2l, and higher scores indicate the worse quality of sleep. Liu et al. introduced it in China in 1996 and proposed that the PSQI was suitable for sleep quality evaluation research in China because of its simplicity, high reliability, and validity, with Cronbach's alpha coefficients of 0.84.
(7) DS14: As a standard assessment tool for distressed personality, the Type D Personality Scale is simple and easy to operate. It contains two dimensional characteristics: negative affect (NA) and Social Inhibition (SI). When the scores of NA and SI are >10, it means a higher level of negative emotions such as anxiety, irritability, and pessimism and a tendency to inhibit the expression of negative emotions in social interaction (36).
(8) HADS: The HADS is a 14-item scale with two subscales measuring anxiety and depression on a four-point (0–3) Likert scale. Scores range from 0 to 21 on both scales. A score above 11 (cut-off threshold) indicates a clinical diagnosis of anxiety and depression. The internal consistency reliability coefficient was adequate for both anxiety (Cronbach's alpha coefficient = 0.72) and depression subscales (Cronbach's alpha coefficient = 0.82), indicating good reliability (37).
(9) SCSQ: The SCSQ was compiled by Xie (38) and included 20 items divided into two dimensions: positive coping and negative coping. The positive coping dimension consists of items 1–12, and the negative coping dimension consists of items 13–20. The questionnaire was self-rated with a four-level Likert scale ranging from “do not use” to “often use,” with 0–3 points each. The results were divided into positive coping scores and negative coping scores. The higher the score of positive coping, the more inclined the respondents were to adopt a positive coping style. The higher the negative response score is, the more inclined the respondents are to adopt a negative coping style (39).

#### CPAP adherence evaluation

In this study, we assessed data from a large cloud database (AirView™) of the continuous positive airway pressure (CPAP) management system to examine adherence to therapy from March 2019 to May 2022. Patient adherence and therapy data are uploaded daily, typically within 1 h after each therapy session. Adherence typically refers to the consistency with which a patient uses PAP therapy. Mean daily use (hours) on all days = total hours of PAP used/total number of follow-up days. Mean daily use on days PAP used = total hours of PAP used/total days used during follow-up. The most widely recognized criterion for adherence is the usage of a PAP device for ≥4 h per night on at least 70% of nights (10). In our study, we extracted the mean daily use of CPAP in 1 week, 1 month, and 3 months, and plotted as continuous changing trajectories. Based on this, we could classify and further understand the subtypes of CPAP adherence.

### Statistical analysis

Data for continuous variables as presented were compared across the three clusters using the ANOVA *F*-test or Kruskal–Wallis test, depending on the distribution of data. Proportions are presented as percentages and were compared with the χ^2^-test. The relationship between putative predictive variables and subsequent adherence clusters was assessed using multinomial logistic regression. Odds ratios (OR) of subgroup membership were estimated for each predictor. Such analysis aims to predict the dependent variables of subtypes (in this case, adherent/non-adherent/improvers) on the basis of categorical or continuous independent variables. All tests were conducted using the Statistical Package for the Social Sciences (SPSS) version 24.0 (SPSS, Chicago, IL, USA). We also describe the trend curve of adherence over time. The present study employed growth mixture models (GMMs) to determine distinct, homogeneous, and longitudinal trajectories in the use of PAP over the 3 months of follow-up. The advantage of GMM is to probabilistically identify homogenous subgroups within larger heterogeneous memberships and represent unobserved heterogeneity by inferring each individual's membership to latent classes from the growth model data (40). MPlus v.8.0 (Los Angeles, CA) was used for all GMM analyses. The model fit between 1 and 4 trajectories was compared (k vs. k-1 trajectories) using the Lo-Mendell Rubin adjusted likelihood ratio test (LRT, *p*<0.05), Parametric Bootstrapped Likelihood Ratio Test (BLRT, *p* < 0.05), Bayesian Information Criteria (BIC), Akaike information criterion (AIC), and convergence (entropy closest to 1.0).

## Results

### Characterization of longitudinal patterns of CPAP users

**Figure 1:**
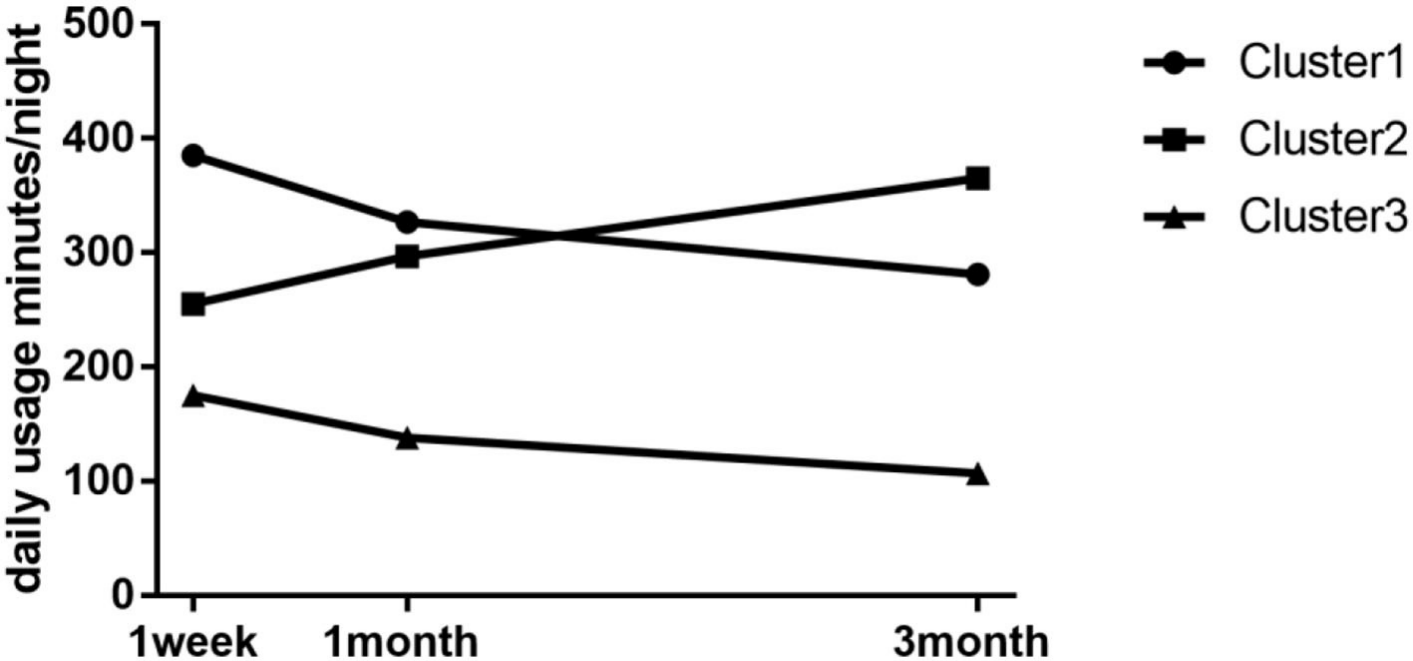
Patterns of longitudinal adherence to CPAP during 3 months treatment. Cluster 1 = adherers; Cluster 2 = improvers; Cluster 3 = non-adherers.

A total of 301 diagnosed patients with OSA who used CPAP regularly were recruited in this study. Growth Mixture Models were evaluated with the number of possible classes ranging from one to four to identify the longitudinal trajectories over time. We used the average daily usage time as the evaluation indicator. Fit indexes for all models are presented in Table 1. Across all models, entropy values ranged from 0.76 to 0.85, indicating a good fit to the data in two to four cluster solutions. The AIC, BIC, and aBIC were observed to decrease as the number of clusters extracted increased, suggesting that a greater number of clusters fit the data progressively better. The LRT test, however, suggested that the three-cluster solution was the best-fitting model, as it was shown to perform significantly better than the four-cluster solution (*p* < 0.001 in cluster 3 and *p* = 0.59 in cluster 4). Therefore, the three-cluster solution was selected as the best representation of the data. The three subgroups identified were labeled “non-adherers,” “improvers,” and “adherers” based on their means of adherence indicators and variation tendency characteristics. The “non-adherers,” “improvers,” and “adherers” subgroups comprised 27.6% (*n* = 83), 18.6% (*n* = 56), and 53.8% (*n* = 162) of the sample, respectively. Trajectories of means on the three adherence variables were determined using minutes on the CPAP every night. Cluster 1 showed high adherence in the 1st week and decreased slightly during the follow-up period and was categorized as the high adherence group (adherers; I = 385, S = −51); cluster 2 presented lower adherence in the 1st week compared with cluster 1 but showed a gradual increase in the following time, so it was the subtypes with improved adherence (improvers; I = 256, S = 50); cluster 3 had low adherence in the 1st week and continued to decline during the follow-up period and was named the low adherence group (non-adherers; I = 176, S = −31; see Figure 1). A comparison of the usage data (nightly use in minutes, the proportion of good adherence, percentage of nights with ≥4 h usage) and therapy issues (e.g, therapeutic pressure, mask leak, residual AHI) among groups was presented in Table 2. The proportion of days compliant and usage time per night at 3 months was 68.8%, 281.4 min; 73.8%, 365.0 min, and 25.7%, 107.7 min, respectively. All groups showed significant improvements after CPAP treatment, and there was no significant difference in residual AHI (2.5 ± 1.8 vs. 2.4 ± 1.8 vs. 2.6 ± 2.0 events/h, *p* = 0.92; see Table 2).

**Table 1:** Fit indices for growth mixture modeling analysis.

						***P***	
**Method**	**Cluster**	**AIC**	**BIC**	**aBIC**	**Entropy**	**LRT**	**BLRT**
GMM	1	9,958.16	9,976.12	9,960.27	/	/	/
	2	9,825.98	9,854.71	9,829.34	0.76	0.00	0.00
	3	9,789.48	9,839.75	9,795.36	0.84	0.00	0.00
	4	9,819.14	9,858.64	9,823.76	0.85	0.59	0.59

AIC, Akaike information criterion; BIC, Bayesian information criterion; aBIC, sample-size-adjusted BIC; LRT, Lo-Mendell Rubin adjusted likelihood ratio test; BLRT, Parametric Bootstrapped likelihood ratio test ratio test.

**Table 2:** Comparison of treatment effect and adherence of three subtypes.

	**1 week**					**1 month**					**3 months**				
	**Cluster 1**	**Cluster 2**	**Cluster 3**	***F*/*X*^2^**	***p***	**Cluster 1**	**Cluster 2**	**Cluster 3**	***F*/*X*^2^**	***p***	**Cluster 1**	**Cluster 2**	**Cluster 3**	***F*/*X*^2^**	***p***
	***N* = 162**	***N* = 56**	***N* = 83**			***N* = 162**	***N* = 56**	***N* = 83**			***N* = 162**	***N* = 56**	***N* = 83**		
Proportion of good compliance, %	149 (92.0)	22 (39.3)	14 (16.9)	144.9	0.00	111 (68.5)	20 (35.7)	2 (2.4)	99.0	0.00	88 (54.7)	40 (71.4)	0 (0.00)	91.7	0.00
Proportion of days compliant (usage ≥4 h/night), %	87.1 ± 15.2	59.4 ± 19.2	40.5 ± 20.5	203.0	0.00	75.9 ± 18.4	64.9 ± 16.9	30.9 ± 16.2	179.0	0.00	68.8 ± 19.2	73.8 ± 12.3	25.7 ± 15.6	201.0	0.00
Device usage (used days), mins/night	391.7 ± 74.6	317.5 ± 73.9	241.0 ± 76.9	111.0	0.00	362.7 ± 68.0	323.7.2 ± 76.3	217.2 ± 79.5	109.0	0.00	332.4 ± 72.7	388.4 ± 57.6	185.2 ± 88.6	150.0	0.00
Daily usage (all days), mins/night	385.1 ± 62.7	255.8 ± 68.9	175.5 ± 53.7	337.6	0.00	327.9 ± 78.6	293.2 ± 72.7	138.4 ± 55.4	202.0	0.00	281.4 ± 76.7	365.0 ± 71.4	107.7 ± 54.4	266.0	0.00
95% pressure, cm H_2_O	10.9 ± 1.8	11.2 ± 1.9	10.6 ± 2.2	1.4	0.20	10.9 ± 1.9	10.9 ± 1.8	10.6 ± 1.9	0.5	0.50	10.9 ± 1.9	10.7 ± 1.4	10.9 ± 1.9	0.3	0.70
95% mask leak, L/min	17.5 ± 11.4	18.5 ± 10.4	15.4 ± 11.0	0.1	0.90	18.4 ± 11.0	18.7 ± 9.7	16.1 ± 10.6	0.1	0.80	19.1 ± 11.9	20.2 ± 9.7	19.2 ± 10.8	0.4	0.60
Residual AHI, events/hour	3.2 ± 2.7	3.0 ± 2.2	3.2 ± 3.0	0.1	0.95	2.6 ± 2.0	2.6 ± 1.8	2.8 ± 2.2	0.2	0.80	2.5 ± 1.8	2.4 ± 1.8	2.6 ± 2.0	0.1	0.92

### Characteristics of demographics, comorbidities, disease severity, and associations with adherence subtypes

The study sample consisted of 301 individuals. The participants were predominantly male (91.3%) and had a mean age of 44.3 years and a mean BMI of 28.4 kg/m^2^. Descriptive characteristics of the study sample are presented in Table 3. Participants in all CPAP user subgroups were similar in terms of age, sex, BMI, educational background, marital status, smoking, and drinking status. However, economic status (χ^2^ = 25.2, *p* < 0.01) and physical exercise (χ^2^ = 45.7, *p* < 0.01) differed significantly among the subtypes. Participants in cluster 1 and cluster 2 had healthier lifestyles and were significantly more likely to engage in prolonged physical activity than participants in cluster 3 (the percentage of participants who exercised frequently was 25.9% in cluster 1, 23.3% in cluster 2, and 6.0% in cluster 3). In addition, participants who reported better economic conditions were significantly more likely to belong to the “adherers” and the “improvers” (see Table 3).

**Table 3:** Demographic characteristics of three patterns of adherence.

	**Cluster 1**	**Cluster 2**	**Cluster 3**	***F*/*X*^2^**	***p***
	***N* = 162**	***N* = 56**	***N* = 83**		
**Age**	45.3 ± 11.2	43.5 ± 10.6	44.1 ± 12.0	0.65	0.50
**Sex**				2.15	0.35
Male	146 (90.1)	50 (89.3)	79 (95.2)		
Female	16 (9.9)	6 (10.7)	4 (4.8)		
**BMI kg/m** ^**2**^	28.5 ± 3.7	28.7 ± 3.1	28.0 ± 3.6	0.72	0.48
**Education**				4.80	0.27
High school or lower	26 (16)	10 (17.9)	17 (20.5)		
Some college	9 (5.6)	6 (10.7)	10 (12)		
College grad or higher	127 (78.4)	40 (71.4)	56 (67.5)		
**Economic**				25.20	0.00
Lower	31 (19.1)	5 (8.9)	26 (31.3)		
Middle	107 (66)	33 (58.9)	53 (63.9)		
High	24 (14.8)	18 (32.1)	4 (4.8)		
**Medical insurance**				7.60	0.42
Medical insurance for urban employees	116 (71.6)	41 (73.2)	61 (73.4)		
Socialized medicine	36 (22.2)	9 (16.1)	12 (14.4)		
Commercial insurance	10 (6.2)	6 (10.7)	10 (12.2)		
**Marital status**				3.50	0.75
Spinsterhood	23 (14.2)	6 (10.7)	15 (18.1)		
Married	130 (80.2)	48 (85.7)	66 (79.5)		
Divorced	9 (5.6)	2 (3.6)	2 (2.4)		
**Physical exercise**				46.7	0.00
Hardly ever	19 (11.7)	12 (21.4)	33 (39.8)		
Occasionally	101 (62.4)	31 (55.3)	45 (54.2)		
Frequently	42 (25.9)	13 (23.3)	5 (6.0)		
**Smoking**				0.80	0.93
No	97 (59.9)	31 (55.4)	45 (54.2)		
Yes	50 (30.9)	19 (33.9)	29 (34.9)		
Quit smoking	15 (9.3)	6 (10.7)	9 (10.8)		
**Drinking**				1.30	0.85
No	76 (46.9)	24 (42.9)	40 (48.2)		
Yes	83 (51.2)	31 (55.4)	40 (48.2)		
Quit drinking	3 (1.9)	1 (1.7)	3 (3.6)		

BMI, Body Mass Index.

Characteristics of comorbidities and disease severity were also assessed to investigate the potential predictors of trajectories. With reference to disease severity, the groups did not differ significantly on any of the apnea hypopnea index (AHI) or 3% oxygen desaturation index (3% ODI). The mean AHI and 3% ODI in the three clusters were 42.8 ± 34.1 events/h, 40.0 ± 22.7 events/h, 37.4 ± 21.9 events/h with AHI; and 37.7 ± 21.9 events/h, 37.1 ± 21.0 events/h, 33.7 ± 18.6 events/h with 3% ODI. The proportion of comorbidities with OSA in our sample was high with hypertension, hyperlipidemia, and hyperuricemia. The average rate of self-reported hypertension in OSA was 46.8% and 29.2% of those taking medication (see Table 4).

**Table 4:** Characteristics of comorbidities and disease severity among three clusters.

	**Cluster 1**	**Cluster 2**	**Cluster 3**	***F*/*X*^2^**	***p***
	***N* = 162**	***N* = 56**	***N* = 83**		
Mallampati score				6.31	0.39
I	21 (13)	9 (16.1)	10 (12.0)		
II	58 (35.8)	25 (44.6)	29 (34.9)		
III	41 (25.3)	13 (23.2)	16 (19.3)		
IV	42 (25.9)	9 (16.1)	28 (33.8)		
AHI	42.8 ± 34.1	40.0 ± 22.7	37.4 ± 20.5	0.92	0.38
ODI3	37.7 ± 21.9	37.1 ± 21.0	33.7 ± 18.6	1.00	0.35
Mean SpO_2_	92.2 ± 2.7	92.1 ± 2.5	92.5 ± 2.7	0.41	0.61
Lowest SpO_2_	74.8 ± 9.5	73.8 ± 9.7	75.1 ± 8.5	0.32	0.68
Hypertension	78 (48.1)	22 (39.3)	41 (46.8)	1.61	0.45
Hyperlipidemia	48 (29.6)	12 (21.4)	20 (24.1)	1.72	0.41
Heart disease	7 (4.3)	1 (1.8)	2 (2.4)	1.13	0.52
Stroke	2 (1.2)	0 (0)	3 (3.6)	3.01	0.17
COPD	2 (1.2)	2 (3.6)	2 (2.4)	1.20	0.62
Diabetes	16 (9.9)	4 (7.1)	5 (6.0)	1.10	0.56
Thyroid disease	12 (7.4)	2 (3.6)	6 (7.2)	4.11	0.38
Hypertension medication	45 (27.8)	14 (25)	29 (34.9)	4.91	0.28
Rhinitis	16 (9.9)	6 (10.7)	9 (10.8)	0.17	0.90
Hyperuricemia	29 (17.9)	14 (25.0)	26 (31.3)	5.70	0.06
High frequency of snoring	119 (73.5)	45 (80.4)	55 (66.3)	4.40	0.62
Loud snoring	119 (73.5)	44 (78.6)	58 (69.9)	3.80	0.70

AHI, Apnea–hypopnea index; ODI3, 3% Oxygen desaturation index; SpO_2_, Oxygen saturation; COPD, Chronic obstructive pulmonary disease.

### Sleep-related health issues, psychological evaluation, and associations with adherence subtypes

Subjective severity of OSA measured by self-reported ESS, ISI, SWIFT, and PSQI as well as OSA-related quality of life measured by FOSQ-10 and QSQ were assessed to explore sleep-related health issues across multiple groups. The longitudinal patterns of adherence were closely related to the degree of daytime sleepiness and daytime function in patients with OSA. The SWIFT scores were significantly increased in cluster 2, and the sleepiness dimensions of the QSQ were decreased in cluster 2, indicating that patients in cluster 2 had a higher level of sleepiness. For the SWIFT, the self-reported scores of “improvers” were significantly higher (12.1 ± 7.0) than “adherers” (8.1 ± 6.0) and “non-adherers” (8.0 ± 6.1; χ^2^ = 9.13, *p* = 0.01), and sleepiness improved the most after 1 month of treatment in “improvers” (with a total change of 3.3 in clusters 1 and 5.6 in cluster 2 and 0.7 in cluster 3, χ^2^ = 7.80, *p* = 0.01). A similar trend was observed for sleepiness dimensions of the QSQ, with “improvers” presenting lower scores on the excessive daytime sleepiness (EDS) dimension in QSQ (4.1 ± 1.1) than “adherers” (4.6 ± 1.2) and “non-adherers” (4.4 ± 1.2) patients (χ^2^ = 3.87, *p* = 0.05). Except for daytime sleepiness, diurnal function also differed on any scores of FOSQ-10 or QSQ total. In the “improvers” group, the participants tended to have more complaints of poor daytime functioning, such as lacking concentration during work or taking more effort to carry out the daily tasks. The FOSQ-10 and QSQ scores were 15.1 ± 4.2 and 4.1 ± 1.0, respectively, in the “improvers” group, which were lower than those in the other two groups. In particular, scores on the diurnal symptom dimension of the QSQ were significantly lower in cluster 2 (4.6 ± 1.6 in cluster 1 vs. 3.8 ± 1.6 in cluster 2 vs. 4.2 ± 1.8 in cluster, χ^2^ = 3.9, *p* = 0.02), showing significant diurnal functional impairment in “improvers.” In addition, “improvers” had poor sleep quality as measured by the PSQI, while insomnia symptoms did not show any difference (see Table 5).

**Table 5:** Sleep-related health issues at baseline and 1 month among three clusters.

	**Cluster 1**	**Cluster 2**	**Cluster 3**	***F*/*X*^2^**	***p***
	**(*N* = 162)**	**(*N* = 56)**	**(*N* = 83)**	
**ESS**					
Baseline	11.5 ± 5.2	12.5 ± 5.5	10.3 ± 4.8	2.62	0.07
1 month	8.1 ± 4.6	10.2 ± 4.7	9.7 ± 4.5	4.85	0.00
Change	3.3 ± 4.9	2.2 ± 4.0	0.5 ± 2.5	1.26	0.28
**FOSQ-10**					
Baseline	15.6 ± 3.0	15.1 ± 4.2	15.2 ± 3.7	3.20	0.76
1 month	17.0 ± 3.4	16.7 ± 2.1	16.1 ± 5.4	8.20	0.09
Change	−1.4 ± 2.2	−1.6 ± 2.4	−0.8 ± 2.5	1.44	0.23
**PSQI**					
Baseline	6.5 ± 2.8	7.5 ± 2.8	6.2 ± 2.5	1.45	0.07
1 month	4.9 ± 2.5	6.1 ± 2.6	5.7 ± 2.9	1.76	0.55
Change	1.5 ± 2.5	1.3 ± 2.1	0.5 ± 1.7	3.48	0.03
**ISI**					
Baseline	10.1 ± 5.4	10.8 ± 5.0	10.5 ± 6.1	0.14	0.78
1 month	6.7 ± 4.6	7.5 ± 4.9	8.4 ± 5.3	2.58	0.07
Change	3.4 ± 4.7	3.2 ± 4.3	2.1 ± 4.4	1.4	0.24
**SWIFT_Total**					
Baseline	8.1 ± 6.0	12.1 ± 7.0	8.0 ± 6.1	9.13	0.01
1 month	4.8 ± 4.7	6.5 ± 5.1	7.2 ± 5.2	6.10	0.03
Change	3.3 ± 6.9	5.6 ± 6.6	0.7 ± 5.8	7.80	0.01
**SWIFT_A**					
Baseline	3.1 ± 3.0	5.2 ± 3.7	3.2 ± 2.7	9.10	0.01
1 month	1.6 ± 2.4	3.4 ± 3.3	3.6 ± 3.1	14.7	0.01
Change	1.5 ± 3.4	1.8 ± 3.9	−0.42 ± 2.4	9.22	0.00
**SWIFT_B**					
Baseline	4.9 ± 3.2	6.7 ± 3.6	4.7 ± 3.3	9.10	0.01
1 month	3.1 ± 3.1	3.0 ± 2.9	3.6 ± 3.5	14.7	0.01
Change	1.7 ± 4.3	3.7 ± 3.7	1.1 ± 4.7	9.22	0.00
**QSQ_Total**					
Baseline	4.6 ± 1.2	4.1 ± 1.0	4.4 ± 1.2	3.03	0.05
1 month	5.1 ± 1.0	4.3 ± 0.9	4.4 ± 0.9	17.2	0.00
Change	−0.5 ± 1.3	−0.2 ± 1.2	0.0 ± 1.2	3.80	0.02
**QSQ_EDS**					
Baseline	4.6 ± 1.2	4.1 ± 1.1	4.4 ± 1.2	3.87	0.05
1 month	5.0 ± 1.5	5.0 ± 1.4	4.8 ± 1.4	0.52	0.59
Change	−0.6 ± 1.9	−0.8 ± 1.9	−0.4 ± 1.8	1.22	0.29
**QSQ_DaySym**					
Baseline	4.6 ± 1.6	3.8 ± 1.6	4.2 ± 1.8	3.9	0.02
1 month	5.2 ± 1.9	3.9 ± 2.1	4.1 ± 1.9	9.96	0.00
Change	−0.6 ± 2.3	−0.1 ± 2.4	0.1 ± 1.9	1.852	0.15
**QSQ_NightSym**					
Baseline	4.2 ± 1.1	3.7 ± 1.2	4.0 ± 1.2	1.80	0.15
1 month	5.0 ± 1.2	3.9 ± 1.4	4.1 ± 1.4	17.10	0.00
Change	−0.8 ± 1.5	−0.2 ± 1.6	−0.1 ± 1.5	5.82	0.03
**QSQ_Emotion**					
Baseline	5.2 ± 1.3	4.6 ± 1.1	5.1 ± 1.4	2.93	0.05
1 month	5.6 ± 1.6	3.9 ± 2.0	4.9 ± 1.8	17.50	0.00
Change	−0.4 ± 1.8	0.7 ± 2.2	0.2 ± 1.8	6.336	0.02
**QSQ_Social**					
Baseline	4.6 ± 1.4	4.4 ± 1.2	4.6 ± 1.6	0.39	0.67
1 month	5.3 ± 1.5	4.9 ± 1.4	4.8 ± 1.7	3.10	0.04
Change	−0.7 ± 1.9	−0.5 ± 1.7	−0.1 ± 2.1	1.66	0.19

ESS, Epworth Sleepiness Scale; FOSQ-10, Functional Outcomes of Sleep Questionnaire-10; PSQI, Pittsburgh Sleep Quality Index; ISI, Insomnia Severity Index; SWIFT, Sleepiness-Wakefulness Inability and Fatigue Test; SWIFT_A, Sleepiness dimension of SWIFT; SWIFT_B, Fatigue dimension of SWIFT; QSQ, Quebec Sleep Questionnaire; QSQ_EDS, Excessive daytime sleepiness dimension of QSQ; QSQ_DaySym, Daytime symptoms dimension of QSQ; QSQ_NightSym, Nocturnal symptoms dimension of QSQ; QSQ_Emotion, Emotion dimension of QSQ; QSQ_Social, Sociability dimension of QSQ.

Of the total psychological variables pooled in the analysis, those who suffered from depression or anxiety were more likely to be present in the poor adherence group. In the “non-adherers” group, scores on the depression dimension of HADS, as well as the negative emotion and Social Inhibition dimensions of type D personality, were significantly higher than those of the other two groups (HADS_depression: 12.9 ± 3.8 in cluster 1 vs. 13.7 ± 3.3 in cluster 2 and 14.6 ± 3.5 in cluster 3, χ^2^ = 6.37, *p* = 0.02; Negative Affectivity: 9.0 ± 6.1 in cluster 1 vs. 9.8 ± 5.1 in cluster 2 vs. 11.5 ± 6.3 in cluster 3, χ^2^ = 4.55, *p* = 0.01; Social Inhibition: 9.3 ± 6.1 in cluster 1 vs. 10.3 ± 5.1 in cluster 2 vs. 11.7 ± 6.5 in cluster 3, χ^2^ = 4.44, *p* = 0.01). In addition, there was an interesting finding about the relationship between coping styles and adherence. Regarding coping styles, participants in “improvers” had high scores of positive coping styles, revealing that they tend to take a positive attitude to solving and deal with problems in their daily life (Table 6).

**Table 6:** Psychological, personality, and familiar coping characteristics at baseline among three clusters.

	**Cluster 1**	**Cluster 2**	**Cluster 3**	***F*/*X*^2^**	***p***
	***N* = 162**	***N* = 56**	***N* = 83**		
HADS-Depression	12.9 ± 3.8	13.7 ± 3.3	14.6 ± 3.5	6.37	0.02
HADS-Anxiety	10.8 ± 2.9	12.3 ± 3.1	11.7 ± 3.2	5.76	0.04
HADS-Depression	110 (67.9)	44 (78.6)	71 (85.5)	9.50	0.08
HADS-Anxiety	79 (48.8)	38 (67.9)	49 (59.0)	6.80	0.03
Type D personality	47 (29.0)	23 (41.1)	44 (53.0)	13.70	0.01
Negative Affectivity	9.0 ± 6.1	9.8 ± 5.1	11.5 ± 6.3	4.55	0.01
Social Inhibition	9.3 ± 6.1	10.3 ± 5.1	11.7 ± 6.5	4.44	0.01
Positive coping index	1.8 ± 0.5	1.9 ± 0.5	1.7 ± 0.5	3.98	0.02
Negative coping index	1.2 ± 0.4	1.1 ± 0.5	1.2 ± 0.4	0.66	0.51
Positive coping tendency	88 (54.3)	33 (58.9)	36 (43.3)	3.80	0.14

### Regression analysis to identify risk factors for adherence patterns

To determine if the sleep-related health issues and psychological covariates significant in univariate analysis were predictors of longitudinal CPAP adherence profiles at the 3-month follow-up, multinomial logistic regression models were created and analyzed. From our univariate analyses (Tables 5, 6). Covariates with *p* < 0.1 were retained for the multivariate analysis. Variables thought to exert influence on adherence, such as age, sex and AHI were forced as covariates into the multivariate analysis. The participants who reported excessive daytime sleepiness were more likely to belong to the “improvers” than the “non-adherers” subgroup (OR = 1.1, *p* = 0.00) and “adherers” subgroup (OR = 1.0, *p* = 0.00). This suggests that for each one point increase on the SWIFT, participants were 15% more likely to belong to the “improvers” subgroup when compared to the “non-adherers.” Similarly, participants who reported greater improvement in sleepiness symptoms at 1 month were more likely to comply with good adherence (cluster 1 vs. cluster 3: OR = 1.21, *p* = 0.00; cluster 2 vs. cluster 3: OR = 1.24, *p* = 0.00). In addition, daytime dysfunction (lower score) favored an increasing adherence pattern compared to “adherers” (cluster 1 vs. cluster 2: OR = 0.72, *p* = 0.01 with QSQ total, OR = 0.76, *p* = 0.02 with QSQ depressed emotion, OR = 0.74, *p* = 0.02 with QSQ excessive daytime sleepiness). As for psychological covariates, our study found that negative emotions may be predictors of poor adherence. Compared with the “adherers” subgroup, the non-adherence group showed higher levels of depression and anxiety emotions and higher scores of Social Inhibition (cluster 1 vs. cluster 3 in HADS-Depression: OR = 1.14, *p* = 0.01; HADS-Anxiety: OR = 1.10, *p* = 0.02; Negative Affectivity: OR = 1.07, *p* = 0.03; Social-Inhibition: OR = 1.03, *p* = 0.04; see Table 7).

**Table 7:** Multinominal logistic regression for predictors with three patterns.

	**Cluster 1 vs**.		**Cluster 1 vs**.		**Cluster 2 vs**.	
	**cluster 2**		**cluster 3**		**cluster 3**	
	**OR**	***P***	**OR**	***p***	**OR**	***p***
ESS	**1.01**	**0.04**	0.99	0.07	1.02	0.26
PSQI	1.10	0.10	0.93	0.29	**1.10**	**0.02**
SWIFT_Total	**1.01**	**0.00**	1.00	0.90	**1.10**	**0.00**
SWIFT_A	**1.22**	**0.02**	1.06	0.50	1.15	0.16
SWIFT_B	0.97	0.96	0.94	0.47	1.05	0.57
QSQ_Total	**0.72**	**0.01**	1.14	0.31	0.83	0.23
QSQ_Emotion	**0.76**	**0.02**	0.92	0.49	0.82	0.15
QSQ_EDS	**0.74**	**0.02**	1.13	0.29	0.84	0.23
QSQ_DaySym	0.77	0.06	0.891	0.216	0.864	0.18
SWIFT_Total Change	**1.05**	**0.03**	**1.06**	**0.01**	**1.11**	**0.00**
SWIFT_A Change	1.02	0.63	**1.21**	**0.00**	**1.24**	**0.00**
SWIFT_B Change	1.11	0.06	1.03	0.37	**1.15**	**0.02**
QSQ_Total Change	1.24	0.10	**1.42**	**0.01**	0.87	0.41
QSQ_Emotion Change	**1.35**	**0.01**	0.85	0.05	1.14	0.17
QSQ_NightSym change	**1.30**	**0.01**	1.35	0.05	0.96	0.76
PSQI change	0.95	0.51	**1.23**	**0.01**	1.17	0.09
HADS-Depression	1.06	0.14	**1.14**	**0.01**	0.93	0.14
HADS-Anxiety	**1.16**	**0.02**	**1.10**	**0.02**	1.05	0.29
Negative-Affectivity	1.02	0.37	**1.07**	**0.03**	0.95	0.12
Social-Inhibition	1.03	0.25	**1.03**	**0.04**	0.96	0.20
Positive coping index	1.50	0.16	1.62	0.06	2.43	0.07

Bold indicates the dependent predict variables of subtypes.

## Discussion

CPAP adherence has been studied for decades, with studies typically focusing on average nightly adherence over the whole study period, which would lose meaningful information with averaging. Growth mixture models (GMMs) are an excellent tool to identify homogeneous subgroups of larger heterogeneous members of longitudinal data. In our study, three distinct patterns of adherence to CPAP use among adult Chinese patients with OSA in the first 3 months of therapy were identified. In addition, we considered that the feedback interaction based on the CPAP therapy remote management platform may influence the adherence of OSA patients over time, and in our analysis, a unique pattern with increasing adherence was found. We also combined demographic, clinical, self-reported sleep issues and psychological risk factors with the distinct patterns of adherence during the first 3 months of CPAP treatment. Excessive daytime sleepiness and daytime functional impairment may be typical characteristics of the “improvers.” Similar to previous studies, negative emotion is a predictor of poor adherence.

Technological progress in the CPAP industry enables more accurate measurement of adherence, rather than early subjective self-reporting. The traditional interpretation of adherence is only divided into good adherence and poor adherence based on the use duration per night and the proportion of used days (the threshold is 4 h per night and 70% of usage days) (41, 42). As indicated in the introduction, simple classification conceals the individual characteristics of patients with OSA treated with CPAP (13, 15). Adherence is not immutable over time; it is too absolute to judge adherence only by a single node. In the whole treatment cycle, the use of CPAP may change due to changes in the surrounding environment. Several analytic methods have been employed to evaluate the intricacies of adherence behavior. Aloia et al. (13) used a separate time series analysis to make a qualitative judgment to categorize CPAP use patterns. Each individual's time series was graphed, and the authors used these graphs to classify the similar profile into seven groups based on a visual inspection of 1 year of use in 71 participants. Babbin et al. (14) replicated the adherence patterns from this previous study using a time series methodology. On this basis, a nomothetic technique named typology of temporal patterns (TTP) was employed to summarize the original clusters and obtained four distinct subtypes named great users, good users, low users, and slow decliners. Those analyses require repeated measurements at fixed intervals over the first 365 days of PAP treatment. Our approach differed from previous analyses in two ways. First, we incorporated growth mixture modeling to examine patterns in longitudinal data with three repeated measures to identify classes or subgroups within a population, which were empirically derived, not rationally or qualitatively derived; that is, we allowed the GMM statistical algorithm to produce the latent profiles (43). Second, we associated the trajectories with multidimensional disease manifestations of OSA patients, which helped to further understand the potential characteristics behind the complex adherence behavior.

Our GMM model showed varying patterns of CPAP use. Consistent with previous studies that considered CPAP use to be binary, we found typically “adherers” and “non-adherers” (44). First, the adherers showed excellent adherence at the beginning of the treatment; although compliance gradually declined with time, it was still considered fully treated at 3 months. The “non-adherers” only used machines for ~176 min at 1 week, and the treatment time showed a slow downwards trend over time, with only 108 min of usage time at 3 months. In addition, our research also discovered a new cluster with increasing adherence over time. One study clustered CPAP data with the Ward linkage, the DTW dissimilarity and the Dunn index and identified six clusters; one of the clusters was similar to “improvers” in our study. The treatment adherence remained at a moderate level at the initial stage, but it showed a gradual upwards trend at the next time (45). A real-world study described the trajectories of ventilator treatment without external human intervention and divided the trajectories into good, medium, and poor adherence groups, and the overall adherence showed a downwards trend (46). Our research was based on the CPAP treatment remote management platform (47). Physicians regularly assessed the treatment reports on the platform and provided targeted solutions to the problems found. Some patients would take longer usage of CPAP after the problems are solved, thus showing a trend of improving adherence. Therefore, previous studies have declared that adherence could be established at an early stage (48–50). Timely discovery and targeted solutions to problems are crucial for establishing better CPAP treatment behavior at later stages. On the one hand, our research supported the standpoint that early adherence can be adjusted and improved. On the other hand, it would be helpful to identify early adherence change patterns by linking multidimensional characteristics (such as patient demography, disease severity, subjective feelings, emotional state, etc.) with the adherence trajectories. For patients with adherence improvement characteristics, special attention should be given, and active intervention will have ideal effects.

In our study, we systematically evaluated the demographic and clinical covariates to further extend our understanding of the clusters and may assist in identifying sources of difficulty with adherence. Of all the covariates that we included, we did not find that the clusters were varied in age, BMI, medical comorbidities, AHI, oxygen desaturation index and other indicators objectively reflecting the severity of the disease. However, daytime sleepiness, reduced daytime function, depressed mood, and positive coping style could distinguish the clusters. We found that those with daytime sleepiness as well as those with daytime functional impairment were more likely to be improvers than either adherers or non-adherers. In the improvers group, excessive daytime sleepiness and impaired daytime function may affect their normal life, and this group has a strong desire for treatment. In addition, the impaired function of “improvers” enhanced significantly at 1 month. Daytime sleepiness was most significantly improved in cluster 2 in our study, which further promoted their willingness to use the CPAP machine regularly. The overall adherence of the patients in this group was good, although the initial use time was less than that in the adherers group. There may be a problem with the CPAP initiation. Telemedicine platform help physicians solve problems as soon as possible, and adherence showed an upwards trend later. Our results are consistent with previous studies. Daytime sleepiness symptoms are predictors of good adherence (23, 51). In this study, whether in the improvers or adherers group, daytime sleepiness was higher than that in the group with poor adherence. Additionally, in the subsequent emotional and personality analysis, we discovered that the participants in this group had positive response. One study also identified that adherent patients tended to have a more positive attitude and adaptive beliefs in CPAP treatment (44, 52). Improvers who have had CPAP treatment may accommodate the device positively and, as time passes, transition to the adherer clusters. This means that this group has a high subjective treatment intention, thus promoting the formation of their good health behavior.

In the symptom assessment of OSA, daytime function is one of the important indicators to evaluate the therapeutic effect of CPAP in patients with OSA, but studies that include daytime function as a predictor of CPAP adherence are limited (22, 53). In our analysis, daytime functional impairment does not distinguish adherers from non-adherers but does distinguish improvers from other clusters. OSA patients with better daytime function are more likely to become adherers or non-adherers. On the one hand, the group with good daytime function has more energy for regular treatment; on the other hand, the better daytime function may also give patients with poor adherence the illusion of no need for treatment.

It is essential to be aware of the psychological assessments of CPAP patients. The final covariates distinguishing the clusters are psychological factors. The literature regarding depressed mood and CPAP adherence is inconclusive. Some studies conducted in small to moderate samples have found no relationships (52, 54, 55). The significant heterogeneity of the literature limits the interpretation and comparison of results. In particular, inconsistencies in different instruments could also be misleading. Mandy et al. recommended the Hospital Anxiety and Depression Scale (HADS) as a tool for screening anxiety and depression symptoms of OSA patients, as it avoids confounders with other emotions. Similar to Mandy's study, depression was independently associated with adherence (26). Anxiety or depression emotion may be a potential target for clinicians to accurately distinguish people with good or poor adherence to CPAP therapy. Finally, personality and coping style, as two predictors of non-intervention, play an important role in the early identification of the pattern of adherence (56). Type D (distressed) personality is defined as a combination of Negative Affectivity and Social Inhibition. In Anders's study, Type D personality occurred in 30% of the patients with OSA and significantly increased the perceived frequency and severity of side effects, which significantly reduced self-reported adherence to CPAP treatment (57). Our study used objective data from the telemedicine management platform of CPAP treatment to prove the above conclusion. Previous studies have found that different family coping constructs provide important information about a family's ability to accept an OSAS diagnosis and adapt to CPAP treatment (15, 24). To our knowledge, this is the first study to examine the importance of personal coping constructs in OSA and their impact on CPAP adherence. Positive coping styles may play an important role in improving adherence to CPAP treatment.

## Conclusion

In conclusion, we have identified, using growth mixture modeling, three latent clusters of CPAP users. We named the clusters adherers, improvers, and non-adherers. Improver is a new pattern of CPAP treatment adherence with the help of physicians' intervention based on telemedicine management platforms. Additionally, we evaluated the relationship between multidimensional characteristics and CPAP adherence, including socio-demography, disease severity, subjectively reported sleep-related problems, and emotional and psychological characteristics, which are important for identifying different subtypes at the initial stage of treatment. In a practical sense, the source of adherence difficulties can be identified and solved by understanding the meticulous identification of CPAP adherence patterns, which is conducive to further optimizing the adherence management system and improving the effectiveness of CPAP treatment for patients with OSA.

## Limitations

Our study has several limitations. We used a convenient sample of participants who participated in CPAP follow-up clinics within 3 months. Although early adherence to CPAP treatment is crucial, a prospective, longitudinal observation can better extend the generalizability of these findings. Additionally, our sample is representative of patients in China, the conclusions may not be directly applicable to patients with OSA in different countries.

## Data availability statement

The original contributions presented in the study are included in the article/supplementary material, further inquiries can be directed to the corresponding author.

## Ethics statement

This study was approved by the Medical Ethics Committee of Peking University People's Hospital (2015PHB187-01). The patients/participants provided their written informed consent to participate in this study.

## Author contributions

HY collected CPAP data and wrote and reviewed the manuscript. XD and SS designed research plan and analysis strategy. CZ and LX analyzed the data. FH revised the paper. All authors contributed to the article and approved the submitted version.

## Funding

This study was supported by the National Natural Science Foundation of China (Grant No. 82020108001 to FH) and the NSFC (Grant No. 82100104 to LX).

## Conflict of interest

The authors declare that the research was conducted in the absence of any commercial or financial relationships that could be construed as a potential conflict of interest.

## Publisher's note

All claims expressed in this article are solely those of the authors and do not necessarily represent those of their affiliated organizations, or those of the publisher, the editors and the reviewers. Any product that may be evaluated in this article, or claim that may be made by its manufacturer, is not guaranteed or endorsed by the publisher.

## References

[1] Gottlieb DJ Punjabi NM. Diagnosis and management of obstructive sleep apnea: a review. J Am Med Assoc. (2020) 323:1389–400. 10.1001/jama.2020.3514 32286648

[2] Patel SR. Obstructive sleep apnea. Ann Intern Med. (2019) 171:12030. 10.7326/AITC201912030 31791057

[3] Mazzotti DR Keenan BT Lim DC Gottlieb DJ Kim J Pack AI. Symptom subtypes of obstructive sleep apnea predict incidence of cardiovascular outcomes. Am J Respirat Criticalcaremed. (2019) 200:493–506. 10.1164/rccm.201808-1509OC 30764637 PMC6701040

[4] Yeghiazarians Y Jneid H Tietjens JR Redline S Brown DL El-Sherif N. Obstructive sleep apnea and cardiovascular disease: a scientific statement from the American Heart Association. Circulation. (2021) 144:e56–67. 10.1161/CIR.0000000000000988 34148375

[5] Linz D McEvoy RD Cowie MR Somers VK Nattel S Lévy P. Associations of obstructive sleep apnea with atrial fibrillation and continuous positive airway pressure treatment: a review. J Am Med Assoc Cardiol. (2018) 3:532–40. 10.1001/jamacardio.2018.0095 29541763

[6] McEvoy RD Antic NA Heeley E Luo Y Ou Q Zhang X. CPAP for prevention of cardiovascular events in obstructive sleep apnea. N Englandjournalofmed. (2016) 375:919–31. 10.1056/NEJMoa1606599 27571048

[7] Patil SP Ayappa IA Caples SM Kimoff RJ Patel SR Harrod CG. Treatment of adult obstructive sleep apnea with positive airway pressure: an American Academy of sleep medicine systematic review, meta-analysis, and GRADE assessment. J Clin Sleep Med. (2019) 15:301–34. 10.5664/jcsm.7638 30736888 PMC6374080

[8] Patil SP Ayappa IA Caples SM Kimoff RJ Patel SR Harrod CG. Treatment of adult obstructive sleep apnea with positive airway pressure: an American Academy of sleep medicine clinical practice guideline. J Clin Sleep Med. (2019) 15:335–43. 10.5664/jcsm.7640 30736887 PMC6374094

[9] Bakker JP Weaver TE Parthasarathy S Aloia MS. Adherence to CPAP: what should we be aiming for, and how can we get there? Chest. (2019) 155:1272–87. 10.1016/j.chest.2019.01.012 30684472

[10] Sawyer AM Gooneratne NS Marcus CL Ofer D Richards KC Weaver TE. systematic review of CPAP adherence across age groups: clinical and empiric insights for developing CPAP adherence interventions. Sleep Med Rev. (2011) 15:343–56. 10.1016/j.smrv.2011.01.003 21652236 PMC3202028

[11] Kribbs NB Pack AI Kline LR Smith PL Schwartz AR Schubert NM. Objective measurement of patterns of nasal CPAP use by patients with obstructive sleep apnea. Am Rev Respir Dis. (1993) 147:887–95. 10.1164/ajrccm/147.4.887 8466125

[12] Weaver TE Kribbs NB Pack AI Kline LR Chugh DK Maislin G. Night-to-night variability in CPAP use over the first three months of treatment. Sleep. (1997) 20:278–83. 10.1093/sleep/20.4.278 9231953

[13] Aloia MS Goodwin MS Velicer WF Arnedt JT Zimmerman M Skrekas J. Time series analysis of treatment adherence patterns in individuals with obstructive sleep apnea. Ann Behav Med. (2008) 36:44–53. 10.1007/s12160-008-9052-9 18726659

[14] Babbin SF Velicer WF Aloia MS Kushida CA. Identifying longitudinal patterns for individuals and subgroups: an example with adherence to treatment for obstructive sleep apnea. Multivariate Behav Res. (2015) 50:91–108. 10.1080/00273171.2014.958211 26609745

[15] Sampaio R Pereira MG Winck JC. A new characterization of adherence patterns to auto-adjusting positive airway pressure in severe obstructive sleep apnea syndrome: clinical and psychological determinants. Sleep Breath. (2013) 17:1145–58. 10.1007/s11325-013-0814-7 23386372

[16] Wohlgemuth WK Chirinos DA Domingo S Wallace DM. Attempters, adherers, and non-adherers: latent profile analysis of CPAP use with correlates. Sleep Med. (2015) 16:336–42. 10.1016/j.sleep.2014.08.013 25441752

[17] Wang Y Geater AF Chai Y Luo J Niu X Hai B. Pre- and in-therapy predictive score models of adult OSAS patients with poor adherence pattern on nCPAP therapy. Pat Prefer Adher. (2015) 9:715–23. 10.2147/PPA.S83105 26064041 PMC4455858

[18] Sunwoo BY Light M Malhotra A. Strategies to augment adherence in the management of sleep-disordered breathing. Respirology. (2020) 25:363–71. 10.1111/resp.13589 31270925 PMC6940560

[19] Wickwire EM Lettieri CJ Cairns AA Collop NA. Maximizing positive airway pressure adherence in adults: a common-sense approach. Chest. (2013) 144:680–93. 10.1378/chest.12-2681 23918114

[20] Sugiyama A Shiota S Yanagihara M Nakayama H Tsuiki S Hayashida K. The role of long-term continuous positive airway pressure in the progression of obstructive sleep apnoea: a longitudinal cohort study. J Sleep Res. (2021) 30:13374. 10.1111/jsr.13374 34137104

[21] Wickwire EM Smith MT Birnbaum S Collop NA. Sleep maintenance insomnia complaints predict poor CPAP adherence: a clinical case series. Sleep Med. (2010) 11:772–86. 10.1016/j.sleep.2010.03.012 20673741

[22] Mehrtash M Bakker JP Ayas N. Predictors of continuous positive airway pressure adherence in patients with obstructive sleep apnea. Lung. (2019) 197:115–21. 10.1007/s00408-018-00193-1 30617618

[23] Olsen S Smith S Oei TP. Adherence to continuous positive airway pressure therapy in obstructive sleep apnoea sufferers: a theoretical approach to treatment adherence and intervention. Clin Psychol Rev. (2008) 28:1355–71. 10.1016/j.cpr.2008.07.004 18752879

[24] Budhiraja R Kushida CA Nichols DA Walsh JK Simon RD Gottlieb DJ. Predictors of sleepiness in obstructive sleep apnoea at baseline and after 6 months of continuous positive airway pressure therapy. Eur Respirat J. (2017) 50:2017. 10.1183/13993003.00348-2017 29191951 PMC6435030

[25] Morrone E Braghiroli A D'Artavilla Lupo N Carli S Tondo P Trentin R. Anxiety and depressive symptoms on continuous positive airway pressure. Long-term adherence in patients with sleep apnoea syndrome. Minerva medica. (2022) 2022:6. 10.23736/S0026-4806.22.08032-6 35332757

[26] Law M Naughton M Ho S Roebuck T Dabscheck E. Depression may reduce adherence during CPAP titration trial. J Clin Sleep Med. (2014) 10:163–9. 10.5664/jcsm.3444 24532999 PMC3899318

[27] Rosa D Amigoni C Rimoldi E Ripa P Ligorio A Fracchiolla M. Obstructive sleep apnea and adherence to continuous positive airway pressure (CPAP) treatment: let's talk about partners! *Healthcare*. (2022) 10:50943. 10.3390/healthcare10050943 35628081 PMC9141202

[28] Cairns A Wickwire E Schaefer E Nyanjom D. A pilot validation study for the NOX T3(TM) portable monitor for the detection of OSA. Sleep Breath. (2014) 18:609–14. 10.1007/s11325-013-0924-2 24442914

[29] Berry RB Budhiraja R Gottlieb DJ Gozal D Iber C Kapur VK. Rules for scoring respiratory events in sleep: update of the 2007 AASM manual for the scoring of sleep and associated events. Deliberations of the sleep apnea definitions task force of the American Academy of sleep medicine. J Clin Sleep Med. (2012) 8:597–619. 10.5664/jcsm.2172 23066376 PMC3459210

[30] Sateia MJ. International classification of sleep disorders-third edition: highlights and modifications. Chest. (2014) 146:1387–94. 10.1378/chest.14-0970 25367475

[31] Wu S Wang R Ma X Zhao Y Yan X He J. Excessive daytime sleepiness assessed by the Epworth Sleepiness Scale and its association with health related quality of life: a population-based study in China. BMC Public Health. (2012) 812:849. 10.1186/1471-2458-12-849 23039935 PMC3524046

[32] Sangal RB. Evaluating sleepiness-related daytime function by querying wakefulness inability and fatigue: Sleepiness-Wakefulness Inability and Fatigue Test (SWIFT). J Clin Sleep Med. (2012) 8:701–11. 10.5664/jcsm.2270 23243405 PMC3501668

[33] Chung KF Kan KK Yeung WF. Assessing insomnia in adolescents: comparison of Insomnia Severity Index, Athens Insomnia Scale and Sleep Quality Index. Sleep Med. (2011) 12:463–70. 10.1016/j.sleep.2010.09.019 21493134

[34] Chasens ER Ratcliffe SJ Weaver TE. Development of the FOSQ-10: a short version of the Functional Outcomes of Sleep Questionnaire. Sleep. (2009) 32:915–9. 10.1093/sleep/32.7.915 19639754 PMC2706905

[35] Huo H Li WY Liu JH Tian X Zhou EH Chen WD. Simplified Chinese version of the Quebec sleep questionnaire was evaluated for reliability and validity. Chin J Otorhinolaryngol Head Neck Surg. (2011) 46:101–7. 10.3760/cma.j.issn.1673-0860.2011.02.004 21426702

[36] Yu XN Zhang J Liu X. Application of the Type D Scale (DS14) in Chinese coronary heart disease patients and healthy controls. J Psychosom Res. (2008) 65:595–601. 10.1016/j.jpsychores.2008.06.009 19027450

[37] Yang Y Ding R Hu D Zhang F Sheng L. Reliability and validity of a Chinese version of the HADS for screening depression and anxiety in psycho - cardiological outpatients. Compr Psychiatry. (2014) 55:215–20. 10.1016/j.comppsych.2013.08.012 24199886

[38] Xie Y. A preliminary study on the reliability and validity of the simple coping style scale. Chinese J Clin Psychol. (1998) 6:114–5. 10.16128/j.1005-3611

[39] Huang Y Su X Si M Xiao W Wang H Wang W. The impacts of coping style and perceived social support on the mental health of undergraduate students during the early phases of the COVID - 19 pandemic in China: a multi-center survey. BMC Psychiatry. (2021) 21:530. 10.1186/s12888-021-03546-y 34706690 PMC8549419

[40] Masterson Creber R Lee CS Lennie TA Topaz M Riegel B. Using growth mixture modeling to identify classes of sodium adherence in adults with heart failure. J Cardio Vasc Nurs. (2014) 29:209–17. 10.1097/JCN.0b013e3182834191 23416937 PMC3695034

[41] Collen J Lettieri C Kelly W Roop S. Clinical and polysomnographic predictors of short-term continuous positive airway pressure compliance. Chest. (2009) 135:704–9. 10.1378/chest.08-2182 19017888

[42] Weaver TE Maislin G Dinges DF Bloxham T George CF Greenberg H. Relationship between hours of CPAP use and achieving normal levels of sleepiness and daily functioning. Sleep. (2007) 30:711–9. 10.1093/sleep/30.6.711 17580592 PMC1978355

[43] Li H Tucker JD Ma W Kim ES Marley G Kang D. Growth trajectories of peer norms, self-efficacy and condom use behavior among sexually active Chinese men who have sex with men: latent class analysis and growth mixture modeling. AIDS Behav. (2020) 24:854–65. 10.1007/s10461-019-02515-7 31016503 PMC7321809

[44] Wild MR Engleman HM Douglas NJ Espie CA. Can psychological factors help us to determine adherence to CPAP? A prospective study. Eur Respirat J. (2004) 24:461–5. 10.1183/09031936.04.00114603 15358707

[45] Bottaz-Bosson G Hamon A Pépin JL Bailly S Samson A. Continuous positive airway pressure adherence trajectories in sleep apnea: clustering with summed discrete Fréchet and dynamic time warping dissimilarities. Stat Med. (2021) 40:5373–96. 10.1002/sim.9130 34250615

[46] Yi H Shang S Zhang Y Zhang C Xu L Wang J. Study on adherence to positive airway pressure treatment for patients with obstructive sleep apnea using real-world big data in a telemedicine management system. Methods. (2022) 204:92–100. 10.1016/j.ymeth.2022.04.006 35439568

[47] Lankford DA. Wireless CPAP patient monitoring: accuracy study. Telemedicine J e-Health. (2004) 10:162–9. 10.1089/1530562041641264 15319046

[48] Chai-Coetzer CL Luo YM Antic NA Zhang XL Chen BY He QY. Predictors of long-term adherence to continuous positive airway pressure therapy in patients with obstructive sleep apnea and cardiovascular disease in the SAVE study. Sleep. (2013) 36:1929–37. 10.5665/sleep.3232 24293768 PMC3825443

[49] May AM Gharibeh T Wang L Hurley A Walia H Strohl KP. Adherence predictors in a randomized trial of moderate-to-severe OSA enriched with women and minorities. Chest. (2018) 154:567–78. 10.1016/j.chest.2018.04.010 29684316 PMC6130325

[50] Van Ryswyk E Anderson CS Antic NA Barbe F Bittencourt L Freed R. Predictors of long-term adherence to continuous positive airway pressure in patients with obstructive sleep apnea and cardiovascular disease. Sleep. (2019) 42:zsz152. 10.1093/sleep/zsz152 31587046

[51] Campos-Rodriguez F Martinez-Alonso M Sanchez-de-la-Torre M Barbe F. Long-term adherence to continuous positive airway pressure therapy in non-sleepy sleep apnea patients. Sleep Med. (2016) 17:1–6. 10.1016/j.sleep.2015.07.038 26847966

[52] Poulet C Veale D Arnol N Lévy P Pepin JL Tyrrell J. Psychological variables as predictors of adherence to treatment by continuous positive airway pressure. Sleep Med. (2009) 10:993–9. 10.1016/j.sleep.2009.01.007 19332381

[53] Somiah M Taxin Z Keating J Mooney AM Norman RG Rapoport DM. Sleep quality, short-term and long-term CPAP adherence. J Clin Sleep Med. (2012) 8:489–500. 10.5664/jcsm.2138 23066359 PMC3459193

[54] Stepnowsky CJ Jr Bardwell WA Moore PJ Ancoli-Israel S Dimsdale JE. Psychologic correlates of compliance with continuous positive airway pressure. Sleep. (2002) 25:758–62. 10.1093/sleep/25.7.758 12405612

[55] Lewis KE Seale L Bartle IE Watkins AJ Ebden P. Early predictors of CPAP use for the treatment of obstructive sleep apnea. Sleep. (2004) 27:134–8. 10.1093/sleep/27.1.134 14998250

[56] Maschauer EL Fairley DM Riha RL. Does personality play a role in continuous positive airway pressure compliance? Breathe. (2017) 13:32–43. 10.1183/20734735.014916 28289449 PMC5343728

[57] Broström A Strömberg A Mårtensson J Ulander M Harder L Svanborg E. Association of Type D personality to perceived side effects and adherence in CPAP-treated patients with OSAS. J Sleep Res. (2007) 16:439–47. 10.1111/j.1365-2869.2007.00620.x 18036091

